# London Letter

**Published:** 1879-06

**Authors:** C. T. Parkes

**Affiliations:** London


### Foreign Correspondence.

---

ARTICLE XIV.

LONDON, April 26, 1879.

*Messrs. Editors:* — Even great London, with its stirring millions, its thousands distressed, diseased and halt and lame, fails to furnish much, or even anything, other than the common ills flesh is heir to. The question how best to relieve these ills, is here answered in terms so often heard and hence so familiar that one almost doubts whether his feet are not resting upon the New World. Of course individualities are seen here and there, in these men and in the things they do, even to the care shown the afflicted under their charge; still, it is mostly an oft-repeated story. Living encyclopædias of the pathological, etiological and symptomatological in the history of every disease and injury, it yet rather surprises one that few, very few, positive assertions come from these men. They seem to be troubled with doubts, although filled to overflowing; yet more is wanted and sought after. Things go contrariwise so often; happenings fail to bear out suppositions in provokingly high ratios. Chaff is more plenty than wheat, in volume if not in weight and worth. Great richness of experience, the outgrowth of familiarity with multitudes of cases, fails to confer infallibility even on the most acute men, either as operators or diagnosticians. Operative blunderings and mistaken diagnoses are things not unheard of even 3,000 miles away from home, and certainly it is somewhat of a comfort to be able to think and say that as yet nothing has been seen that would induce me to speak in the least degree disparagingly of my countrymen on either score. High water mark measures no further up on the walls of stone here than it does within rifle range of my own door. "Good cheer, brethren," be our motto. "*Toujours de l'Audacité!*" I believe the quotation is French; my proximity to that nation is ample excuse for its use, and besides, when written so, the motto is robbed of some of its boldness.

Some ten or twelve years ago, the late Professor Freer enunciated a principle of procedure in the formation of a gastric fistula in dogs for class experimentation, which he said must be carefully carried out in order that the operation might be successful. This important element of safety consisted in so doing the operation as to provide for firm and extensive adhesions between the stomach and abdominal walls, before any opening is made into the stomach itself. Prof. Freer always made application in his teachings of the same course of procedure, which ought to be carried out, he said, with minute care, in all operations of a like nature done upon the human body.

The operation for making a permanent opening into the stomach, called gastrostomy, done to prolong life and relieve the agony of patients suffering from closure of the œsophagus as the result of disease, or the mere opening of it for the removal of foreign bodies, is recommended as a proper surgical procedure, and has been done a number of times — still, to Mr. Howse, of Guy's Hospital, is certainly due the credit of demonstrating its absolute feasibility, comparative safety and evident necessity.

Mr. Howse considers that the uniformly successful result following his efforts so far is mainly due to the special method of procedure which he has followed in all his cases; this manner of operating accomplishes perfectly the very desideratum which Prof. Freer deemed so necessary and urgently advised. The first part of the operation is devoted to and done with the intention of securing a firm wide attachment of the stomach to the abdominal walls. This consists in fastening the two together by ligatures introduced in a special manner and leaving them so attached for five or six days, which is long enough, according to Mr. Howse's experience, to develop firm union; after which the stomach itself is opened. In none of these cases was there any undue inflammation or any other interference with the progress of the case to recovery. The fistula is completed by opening the stomach through the lips of the abdomino-gastric junction — a proper gum elastic tube is introduced into the cavity of the viscus and retained there by strapping, and through this tube the patient can be fed without discomfort or trouble.

Mr. Howse naturally expresses the hope, encouraged as he is by his successes, following the operation, that this procedure will be earlier resorted to in the class of cases for which it is oftenest done — epithelioma of the œsophagus, and in addition that it will come to be looked upon as a necessary and accepted course of treatment in cases of traumatic occlusion or specific stricture. Up to the present time five operations have been done by him, four for closure of the œsophagus by epithelioma and one for traumatic stricture.

Of those done for epithelioma, one lived seven months after the operation and finally died from extension of the cancerous disease to surrounding vital organs. During this long period the patient was entirely free from the agonizing distress incidental to slow starvation — feeding herself comfortably and easily through the tube or external œsophagus. I might even justly say that the feeding was not robbed of enjoyment, for the patient masticated her food and then introduced it into the stomach through the tube. Two others, operated upon for the same disease, lived for weeks and months respectively, and died from epithelioma of the lungs, each deriving benefit from the operation. The fourth one died from renal coma a few days subsequent to the operation. In none of them was there the least discomfort arising from the operation, and the pathological specimens of each case, now preserved in the museum, demonstrate positively that in neither case was there any peritonitis developed from the interference, as is shown by the entire absence of any adhesions other than those sought for and obtained between the stomach and abdominal walls.

Through Mr. Howse's courtesy and kindness to me, I witnessed the operation in the fifth case, and have seen the patient several times during the several weeks following the operation, and can bear witness to the comfortable condition of the patient, the relief afforded her, and to the absolute absence of any local trouble from the wound. The patient had swallowed a quantity of muriatic acid with suicidal intentions some months ago, and, as a consequence of this act, stricture of the œsophagus had gradually developed, defying and resenting all efforts at dilatation. Any such like attempts at relief were followed by severe bleeding and other bad signs, sufficient to make the efforts dangerous. Mr. Howse did the operation partly to avoid expected emaciation from inability to swallow, but mainly to put the parts above absolutely at rest for months, so that all granulating surfaces might become healed over and toleration of dilating instruments follow. If this result came about, well and good; if not, then the patient could live quite comfortably with the new mouth to her stomach — digestion, apparently, being very little affected by the new way of taking food. An oblique incision was made, about 9 Cm. long, parallel to the cartilaginous margin of the chest on the left side, in the epigastric region, far enough away from the cartilage to allow of room for the application of the outside row of stitches used to fasten the stomach to the abdominal walls, and carried down to the rectus abdominis muscle, the fibers of which were the landmark as to position. The under layer of its sheath is the indication of close proximity to the peritoneum. All bleeding vessels were controlled with catgut ligatures. The peritoneum was then carefully opened and the anterior wall of the stomach sought for. Mr. Howse gives three means of recognizing this organ. The mistake of opening the transverse colon has been made, but according to him such an accident should never happen. 1st, the walls of the stomach are much thicker than those of any intestine; 2d, the color is lighter, and there are no longitudinal fibers; 3d, the two layers of the omentum, passing from the stomach to the colon, can be easily recognized. Having reached the stomach, the next step is to stitch it to the abdominal walls, and in this consists the essential part of the operation, and this part must be carefully and accurately done. He employs two rows of sutures. The first introduced at intervals of 1 Cm. from each other all around the margin of the external wound, fully 3½ Cm. from that margin. The second joins the stomach to the edges of the incision, leaving about 1 Cm. of its surface exposed in the wound, as its edges are subsequently drawn together. In passing all of the sutures into the walls of the stomach, great care is taken to prevent the needle entering the cavity of that organ. Great stress is laid upon this matter, for if they do enter, fatal peritonitis is quite sure to follow upon the escape of gas or other matters through even these slight wounds. With the greatest impunity and entire absence of any dread of doing harm, the stomach walls are grasped by the fingers at the points chosen for the introduction of the needle, and in this way the operator absosolutely assures himself that only the peritoneal covering and a portion of the muscular coat is pierced by the needle or included in the ligature. Perhaps a quarter of an inch of tissue is taken up in the grasp of each suture. Of course the needle is first passed through the entire thickness of the abdominal walls at the proper distance from the edge of the entrance wound, then through the stomach with the care specified, then out through the external coverings of the body again, and finally the two ends are tied over a piece of catheter; to give breadth of contact between the two parts and to prevent puckering. All the sutures of the outer row were thus carefully introduced until the entire circuit of the external wound was made; cat-gut was used for these sutures. The edges of the external wound were then united to the stomach in the same careful manner, and finally the wound itself was closed almost entirely by sutures. A silver ligature was introduced into the walls of the stomach to indicate the point at which the knife should be introduced into it, to complete the fistula after sufficient time had elapsed, to be sure that the adhesions desired were sufficiently firm. In all the five cases operated upon, this final opening was safely made; the post mortem examination in those who died showing the union complete and perfect. The feeding tube is then introduced, and all is well. Mr. Howse is a firm believer in the benefits of Mr. Lister's antiseptic dressing, and carries out that method of treatment in all operations, and to its great aid gives the credit of the safety after this rather formidable operation. One of Mr. Howse's colleagues at Guy's, has done the operation twice; once for epithelioma, and once for syphilitic stricture. The first of these died in a few days from general prostration, the operation being done after the old plan, such as is followed in colotomy. The second was done with all the care so earnestly insisted upon and followed out by Mr. Howse, and is now living comfortably with the fistula completely established, five months after the operation. So a great deal of credit must be accorded to the *method* of doing the operation.

Again, Mr. Howse is a skillful, very careful and exceedingly ready operator—absolutely sure of his anatomy in minutiæ—and in these happy possessions, we must recognize great elements of success. You will pardon me for alluding to another somewhat heroic procedure practiced by him in the treatment of omental hernia. A large number of successful cases has convinced him that the best plan of treatment is to cut down upon the protruding mass, and remove it. They always grow longer, predispose to the formation of intestinal hernia, are liable to become strangulated, are never safely controlled by a truss, have never given rise to any trouble when removed under the spray, and the cure is radical. The base is tied with carbolized silk and dropped after the mass is cut away. Two excellent reasons are given: 1st, it is perfectly safe; 2d, it is a sure cure.

Several cases of admirable cure of nævi have come under my notice following treatment by the astringent caustic sodium ethylate. This caustic is made by putting pieces of metallic sodium into a small quantity of absolute alcohol in a wide-necked dish until the bubbles of gas cease to rise, when we have a snow-like, semi-crystallized substance. To this is added about its own bulk of absolute alcohol again, and there results a brownish-colored fluid about like ordinary mucilage, which should be kept in a glass-stoppered bottle fitted with a glass rod for application. It is applied to the surface of the nævoid tissue once a week, or once a month, according as the resulting eschar falls. Its application is accompanied with very slight pain; the resulting cure is all that can be desired; scarcely any evidence of cicatrix or whitening following. Large venous nævi are successfully cured by Mr. Thomas Smith, of St. Bartholomew's, by introducing large strands of yarn, soaked in the per-cholride of iron, through the mass in all directions, by means of a good-sized needle, so that the iron will not be squeezed out of the yarn by the edges of the wound. The yarn is left in some days until free suppuration is excited. By the way, I hope the gentleman last mentioned will forgive me for saying that he is the neatest and most expert operator in general surgery that I have seen in London.

There is at least one gentleman in London who does not believe in doing osteotomy for genu valgum; on the contrary, he thinks such interference uncalled for in children and very seldom necessary at any time of life. This is Mr. Brodhurst of the Royal Orthopedic Hospital, an authority on such questions whose statements are of great value. His first objection is based upon the fact that death has more than once followed as the result of the operation; second, that if no fatal result ensue, anchylosis and loss of joint does occur quite often; third, the deformity is, in many instances, scarcely benefited at all by this hazardous procedure; fourth, what is far more important, perfectly satifactory results and relief of deformity can be obtained in a much simpler way, namely: by subcutaneous division of the tendon of the biceps femoris muscle, and the external lateral ligament of the knee joint. This simple operation is to be followed by the use of side splints, so adapted as to keep the parts in line for a sufficient time. Mr. Brodhurst showed me a girl, 17 years of age, in whose case he adopted this plan of relieving excessive deformity from genu valgum, and the result was perfect in all respects.

He speaks confidently about his own method, and urgently advises against the major operation. There is no deformity incident to shortened tendons or contracted tissue of any kind, but what is relieved at this hospital, no matter what the original cause of the trouble. The skill in operating is unsurpassed, and the subsequent treatment remarkable only for the gentleness in application of restraining appliances. The universal splint is one made of tinned hoop-iron, about 1½ Cm. broad. It is perfectly pliable and can be moulded to any case of talipes, however misshapen; and upon it they rely to gradually bring the parts into proper position after division of tendons. No attempt at extension is made until after the wounds of tenotomy have entirely healed. Their rule is to divide everything that is tight. Varus is treated according to an invariable rule; first, bring the foot into line with the leg by division of the anterior and posterior tibial tendons with the plantar fascia if necessary. No more is done until the cure of this part is secured, then the second part of the treatment is commenced by curing the equinus, after the usual method of dividing the tendo Achillis.

No one can avoid expressing wonder at the marvelous results of treatment in this hospital. I saw less blood from the division of several tendons in twenty different cases, than I have seen follow one case of cutting the tendo Achillis in many instances. The knife used is small in the blade, introduced close to the tendon and beneath it, and the cut made outwards. A probe-pointed knife is used only for the tibialis posticus tendon and the plantar fascia. So satisfactory are the results from the use of the splints above mentioned, they very seldom find it necessary to use special shoes for talipes—I might say never in young children.

I listened to a very valuable paper read before the Clinical Society of London one week ago, on amputation of the hip joint, the principal interest to me centering in the novel method adopted and used to control hæmorrhage. This new and truly novel method has been introduced lately by Mr. Davy. It is done by means of a wooden lever about 60 Cm. long, measuring 2½ Cm. in diameter at either end for 15 Cm. of the length; being only 2 Cm. in diameter for the remainder. It is turned perfectly smooth and is well polished, the ends being slightly rounded. It is introduced into the patient's rectum sufficiently far for the end to rest in the hollow opposite to the sacro-iliac synchondrosis, between the psoas muscles and the base of the sacrum, where it is made to rest upon the common iliac artery, controlling the blood current perfectly; so that not a drop of blood is lost, except while the pressure is relieved to secure the smaller arteries. It has been used four or five times successfully and without any apparent damage whatever. The pressure is made by elevating the outer end, using the anus as a fulcrum. The instrument or lever, is steadied by holding it against the opposite thigh.

I am reminded that this letter has approached the elements of a course of lectures on physiology, so far as the beginning and ending go. We began at the stomach and have concluded as above. That is long enough certainly if measured by feet.

C. T. PARKES.

